# Supplementary material for: Perturbation of de novo lipogenesis hinders MERS-CoV assembly and release, but not the biogenesis of viral replication organelles
Source: J Virol. 2025 Feb 20;99(3):e02282-24. doi: 10.1128/jvi.02282-24 (PMC11915874; doi:10.1128/jvi.02282-24)
Supplement: Supplemental material — Figures S1 to S5 and Table S1. [file jvi.02282-24-s0001.pdf]

SUPPLEMENTAL MATERIAL

Perturbation of *de novo* lipogenesis hinders MERS-CoV assembly and release, but not the biogenesis of viral replication organelles

M. Soultsioti[1], A.W.M. de Jong[2], N. Blomberg[3], A. Tas[1], M. Giera[3], E. J. Snijder[1]#, M. Barcena[2] #

Figure S1

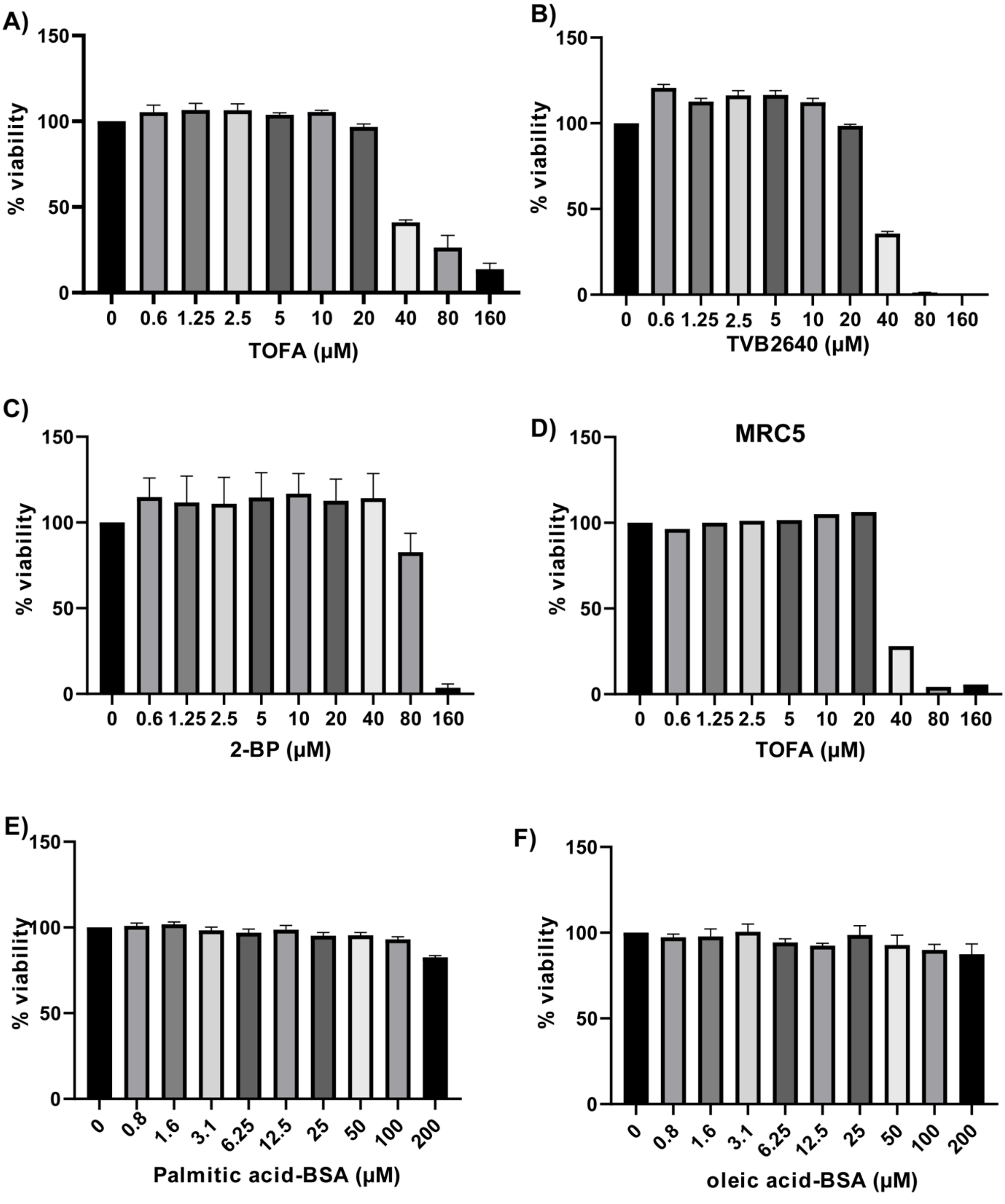

Figure S1. Cytotoxicity assays. Cell viability was evaluated following treatment with various small-molecule inhibitors used in this study in different cell lines using an MTS assay at 24 h post compound treatment. Viability was normalized against the DMSO control. A) TOFA in Huh7 cells, B) TVB-2640 in Huh7 cells, C) 2-BP in Huh7 cells, D) TOFA in MRC5 cells, E) palmitic acid-BSA in Huh7 cells, F) oleic acid-BSA in Huh7 cells.

Figure S2

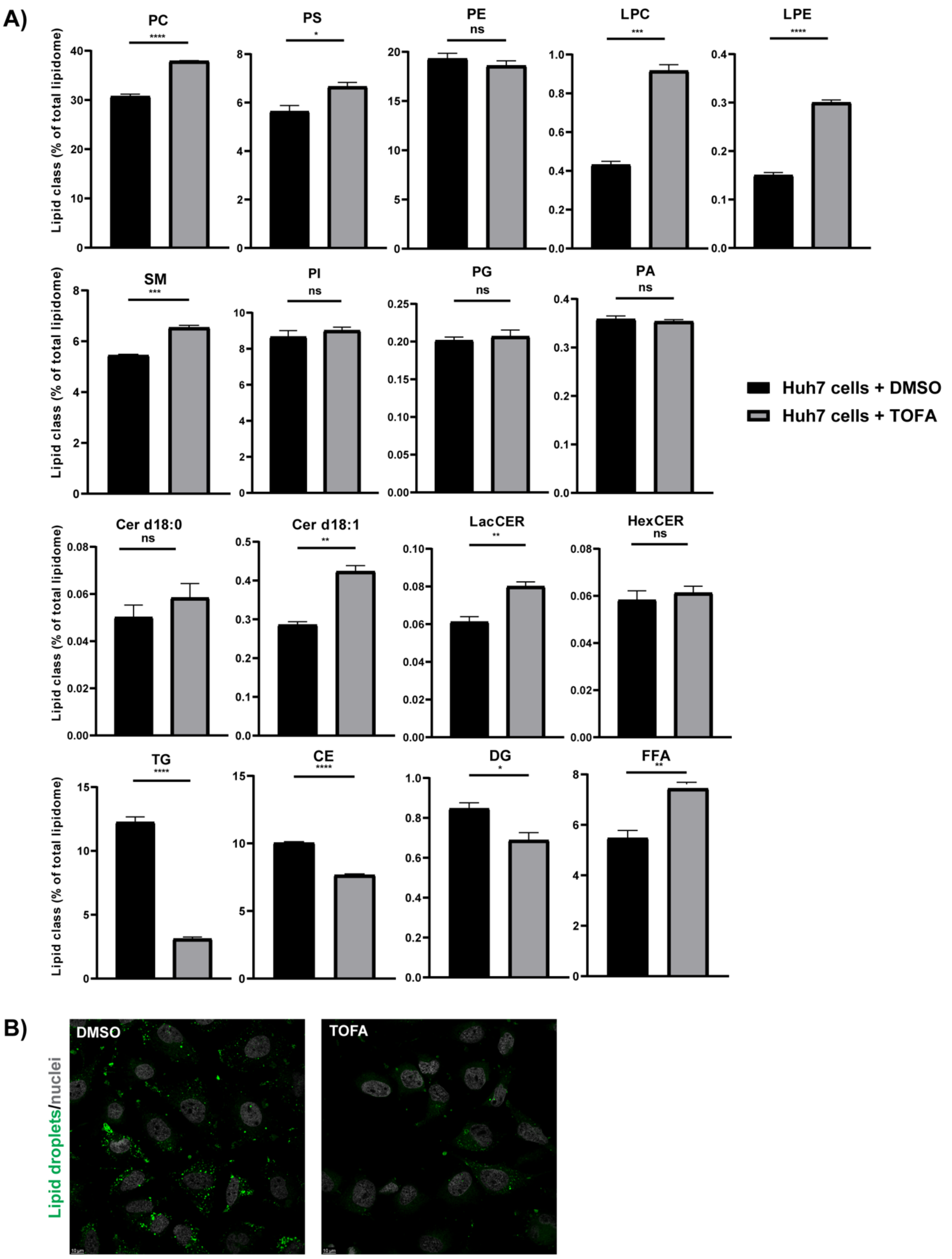

Figure S2. Changes in the cellular lipidomic profile upon inhibition of ACC. Huh7 cells were treated with DMSO or TOFA. The amounts of intracellular lipids were determined by mass spectrometry 12 h after compound addition. Different classes of lipids and their relative abundance compared to the total lipidome are depicted in the graphs. A) phosphatidylcholine lipids (PC), phosphatidylserine (PS), phosphatidylethanolamine (PE), Lyso-PC, Lyso-PE,

sphingomyelins (SM), phosphatidylinositides (PI), phosphatidylglycerides (PG), phosphatidic acids (PA), ceramides (Cer d18:0), Cer 18:1, lactosyl-CER (LacCER), hexosyl-ceramides, triacylglycerols (TG), cholesterol esters (CE), diacylglycerols (DG) and free fatty acids (FFA). Data are represented as mean ± SD of three biological replicates. Statistical significance was calculated using unpaired Student's t-test, where * $p<0.05$, ** $p< 0.01$, *** $p< 0.001$, **** $p< 0.0001$, B) Huh7 cells were treated as described above and 12h post treatment medium was removed, the cells were washed and incubated with BODIPY 493/503 for 1 h. Cells were then fixed, the nuclei were stained with Hoechst 33342 and the presence of lipid droplets was analyzed by confocal microscopy. Representative microscopy images of two independent biological replicates are depicted.

Figure S3

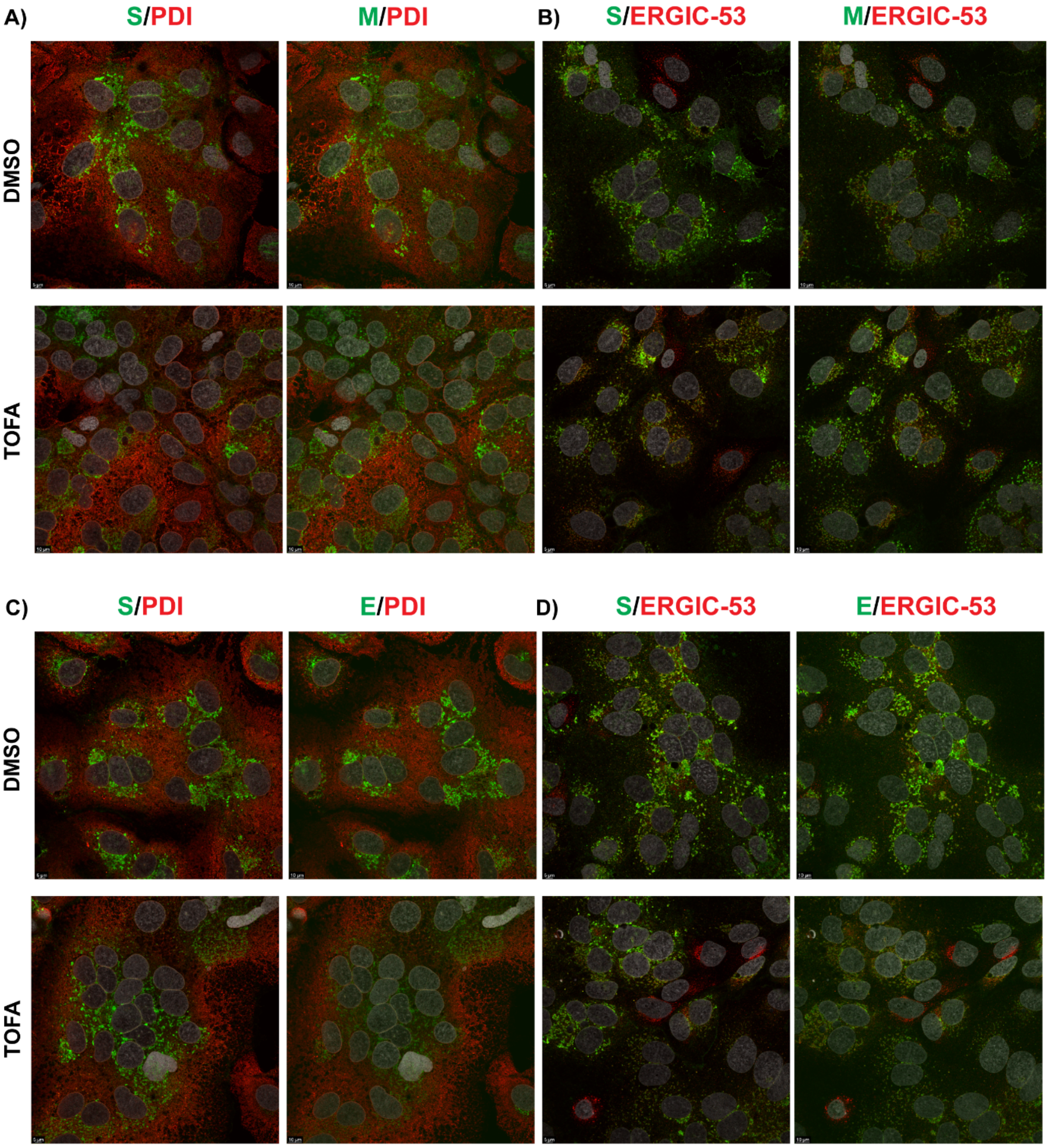

Figure S3. Subcellular localization of MERS-CoV structural proteins in the presence of TOFA**.** Huh7 cells were infected with MERS-CoV and treated with TOFA as previously described and fixed at 12 h p.i. Cells were labelled simultaneously for S (green) and a marker for PDI or ERGIC (red) and M or E (green) in combination with a marker for PDI or ERGIC (red). Confocal images are representative of at least two independent biological replicates.

Figure S4

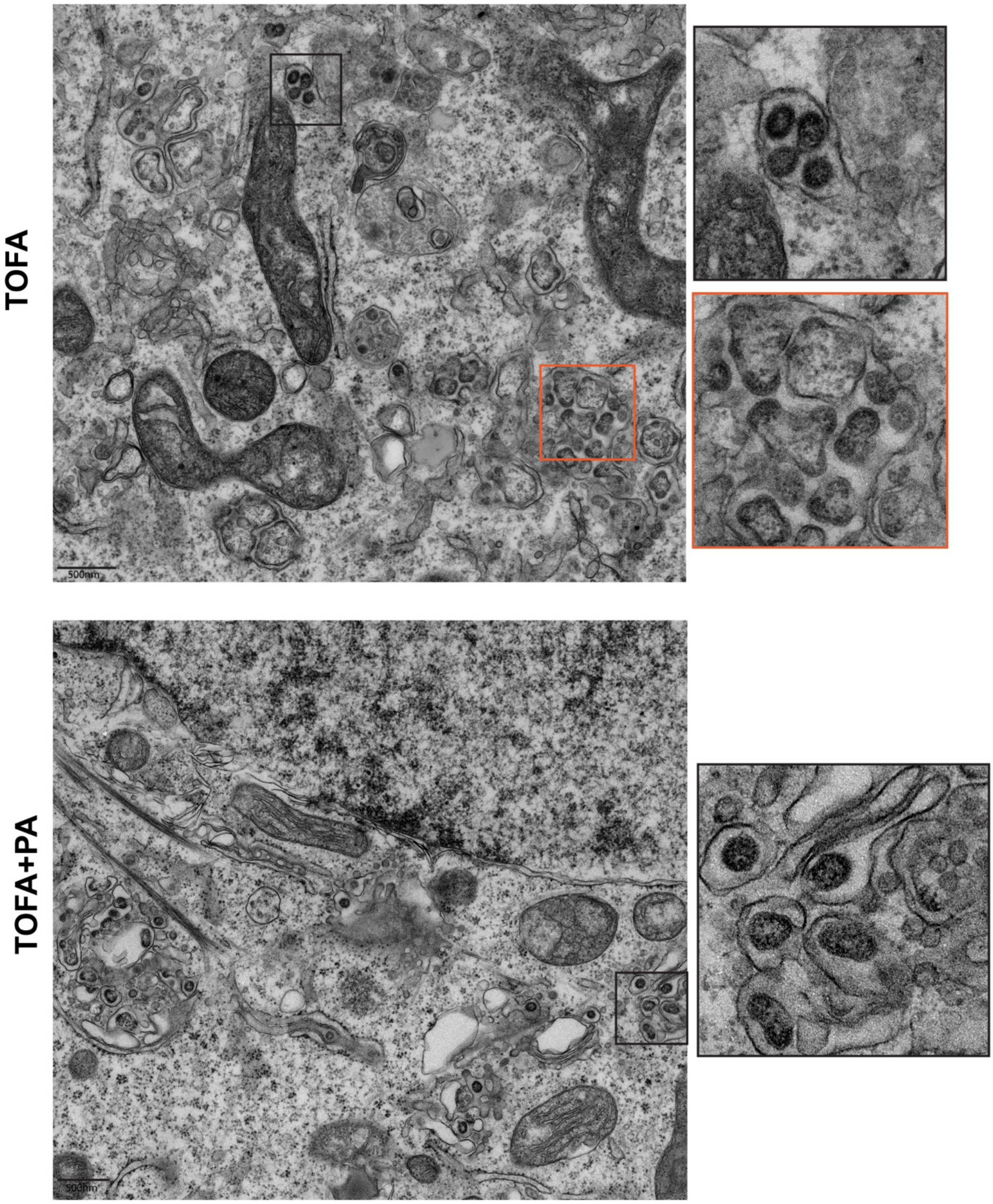

Figure S4. Representative EM images of (early and late) assembly events during MERS-CoV replication and TOFA or TOFA and palmitic acid supplementation used for quantifications for Figure 7G. Scale bar is 500nm.

Figure S5

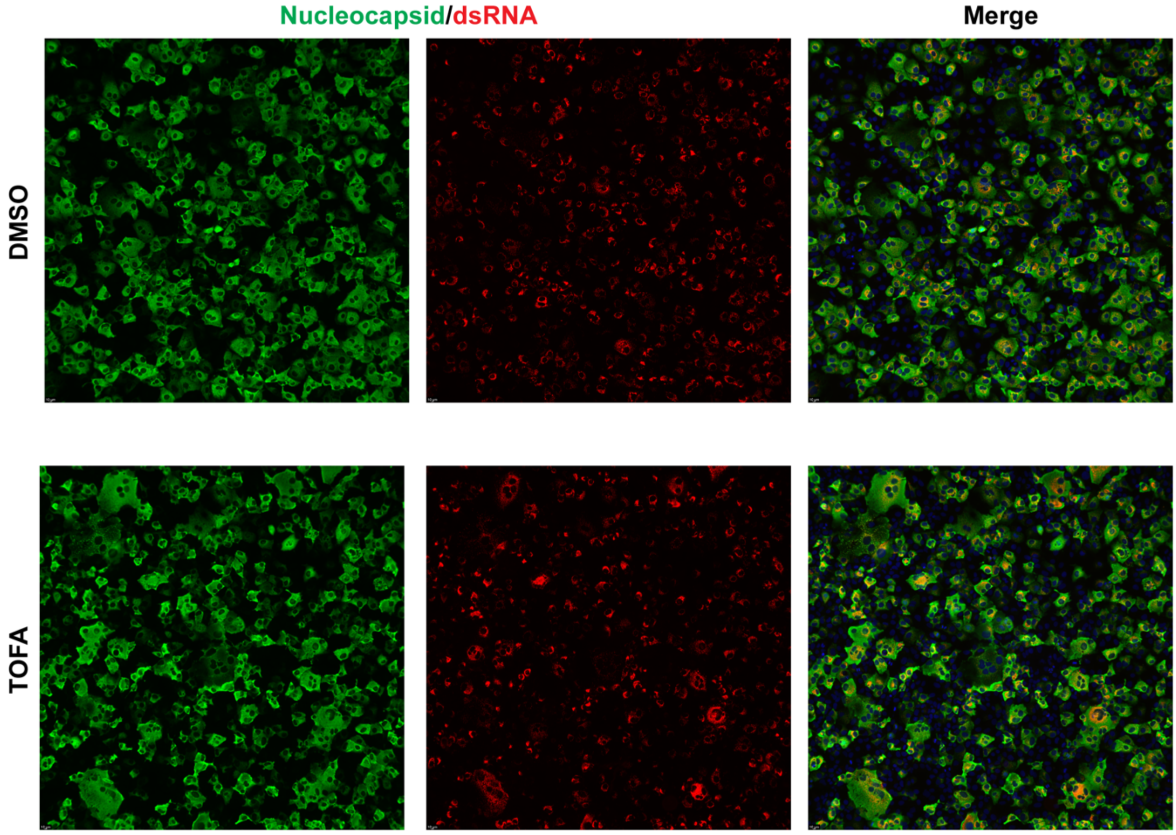

Figure S5. Immunofluorescence analysis of MERS-CoV-infected (MOI 5) Huh7 cells, DMSO- or TOFA-treated, 12 h p.i. Cells were labelled for the N protein (green) and dsRNA (red).

Supplementary Table 1: Primers used in this study

Oligonucleotide name	Sequence (5'-3')	Source reference
ACLY Forward primer	CCTCGAGATCAATCCCCTTGTA	(6)
ACLY Reverse primer	CGATGTCACCCCACTTCACTTT	(6)
ACC Forward primer	GAGGGCTAGGTCTTTCTGGAAG	(6)
ACC Reverse primer	CCACAGTGAAATCTCGTTGAGA	(6)
FASN Forward primer	AGCTGCCAGAGTCGGAGAAC	(6)
FASN Reverse primer	TGTAGCCCACGAGTGTCTCG	(6)
SCD-1 Forward primer	TGCTGCCCACCTCTTCGGATAT	(6)
SCD-1 Reverse primer	TAGTTGTGGAAGCCCTCACCCA	(6)
DGAT1 Forward primer	GGCATCCTGAACTGGTGTGTG	(6)
DGAT1 Reverse primer	GAGCTTGAGGAAGAGGATGGTG	(6)
ADRP Forward primer	GGGATCCCTGTCTACCAAGC	(6)
ADRP Reverse primer	AGATGTCGCCTGCCATCACC	(6)
CPT1a Forward primer	TGAGCGACTGGTGGGAGGAG	(6)
CPT1a Reverse primer	GAGCCAGACCTTGAAGTAGCG	(6)
ACOX Forward primer	TCCTGCCCACCTTGCTICAC	(6)
ACOX Reverse primer	TTGGGGCCGATGTCACCAAC	(6)
human b-actin Forward primer	CACAGAGCCTCGCCTTTGC	(73)
human b-actin Reverse primer	AATCCTTCTGACCCATGCCC	(73)
MERS-CoV N Forward primer	GTACCTCTTAATGCCAATTC	in-house design
MERS-CoV N Reverse primer	GAGCCAGTTGCNTTAATTC	in-house design
MERS-CoV N probe with TexasRed 5' fluorophore	TCTGTCCTGTCTCCGCCAATAC	in-house design
MERS-CoV Nsp2-3 Forward primer	CCGACTCTCTTTAGACTTA	in-house design
MERS-CoV Nsp2-3 Reverse primer	ACAGCATGAATGTTGTAC	in-house design
MERS-CoV Nsp2-3 probe with FAM 5' fluorophore	AACACTTCTTACAGCAGCAACCTC	in-house design
